# IGF-1 protects against angiotensin II-induced cardiac fibrosis by targeting αSMA

**DOI:** 10.1038/s41419-021-03965-5

**Published:** 2021-07-09

**Authors:** Sangmi Ock, Woojin Ham, Chae Won Kang, Hyun Kang, Wang Soo Lee, Jaetaek Kim

**Affiliations:** 1 Division of Endocrinology and Metabolism, Department of Internal Medicine, College of Medicine, Chung-Ang University, Seoul, Korea; 2 Department of Anesthesiology, College of Medicine, Chung-Ang University, Seoul, Korea; 3 Division of Cardiology, Department of Internal Medicine, College of Medicine, Chung-Ang University, Seoul, Korea

**Keywords:** Heart failure, Heart failure

## Abstract

The insulin-like growth factor 1 receptor (IGF-1R) signaling in cardiomyocytes is implicated in physiological hypertrophy and myocardial aging. Although fibroblasts account for a small amount of the heart, they are activated when the heart is damaged to promote cardiac remodeling. However, the role of IGF-1R signaling in cardiac fibroblasts is still unknown. In this study, we investigated the roles of IGF-1 signaling during agonist-induced cardiac fibrosis and evaluated the molecular mechanisms in cultured cardiac fibroblasts. Using an experimental model of cardiac fibrosis with angiotensin II/phenylephrine (AngII/PE) infusion, we found severe interstitial fibrosis in the AngII/PE infused myofibroblast-specific IGF-1R knockout mice compared to the wild-type mice. In contrast, low-dose IGF-1 infusion markedly attenuated AngII-induced cardiac fibrosis by inhibiting fibroblast proliferation and differentiation. Mechanistically, we demonstrated that IGF-1-attenuated AngII-induced cardiac fibrosis through the Akt pathway and through suppression of rho-associated coiled-coil containing kinases (ROCK)2-mediated α-smooth muscle actin (αSMA) expression. Our study highlights a novel function of the IGF-1/IGF-1R signaling in agonist-induced cardiac fibrosis. We propose that low-dose IGF-1 may be an efficacious therapeutic avenue against cardiac fibrosis.

## Introduction

Cardiac fibrosis characterized by excessive synthesis or diminished degradation of extracellular matrix (ECM) proteins is a hallmark of nearly all types of heart disease and contributes to the progression of heart failure [1]. Upon mechanical or biochemical stimulation, resident cardiac fibroblasts become activated and differentiate into α-smooth muscle actin (αSMA)-expressing myofibroblasts, which are critical in the exacerbated secretion of ECM [2, 3]. Multiple signaling pathways have been implicated in the conversion of fibroblasts into myofibroblasts including transforming growth factor β (TGFβ)/mothers against decapentaplegic homolog (Smad), angiotensin II (AngII), endothelin, and wingless-related integration site (Wnt)/β-catenin [1]. Although both angiotensin-converting enzyme inhibitors (ACEIs) and AngII receptor blockers (ARBs) have already shown significant efficacy in reducing cardiac fibrosis in human and animal models of heart failure, they have not been approved for the treatment of cardiac fibrosis [4]. In this context, suppression of myofibroblast differentiation could be an effective therapeutic approach to prevent excessive cardiac fibrosis.

Insulin-like growth factor (IGF)-1 is mainly synthesized by the liver which binds its ubiquitously expressed cognate receptor, IGF-1 receptor (IGF-1R). Studies of cardiomyocyte-specific knockout (KO) or transgenic IGF-1R mice showed that IGF-1R activates phosphoinositide 3-kinase (PI3K)–Akt signaling pathways through its intrinsic tyrosine kinase activity and regulates cardiomyocyte survival, hypertrophy, and aging [5–7]. In addition, some pieces of evidence are suggesting that IGF-1 signaling has beneficial effects against cardiac fibrosis. Cardiac overexpression of IGF-1R prevents diabetes-induced cardiac fibrosis and diastolic dysfunction [8]. Furthermore, the microencapsulated IGF-1 therapy reduces cardiac fibrosis in the porcine acute myocardial infarction model [9]. However, the role of IGF-1/IGF-1R signaling in the development of myocardial interstitial fibrosis under non-ischemic cardiac injury has never been directly addressed.

The present study was undertaken to examine the hypothesis that (1) deletion of IGF-1R in cardiac myofibroblasts causes aggravation of cardiac fibrosis against agonist-induced cardiac injury, and that (2) AngII-induced cardiac fibrosis could be alleviated by IGF-1 administration. Further, we determined underlying mechanisms of the inhibitory effect of IGF-1 in AngII-induced cardiac fibroblast differentiation.

## Results

### IGF-1R expression patterns in mouse hearts and generation of CFIGF1RKO mice

**Fig. 1:**
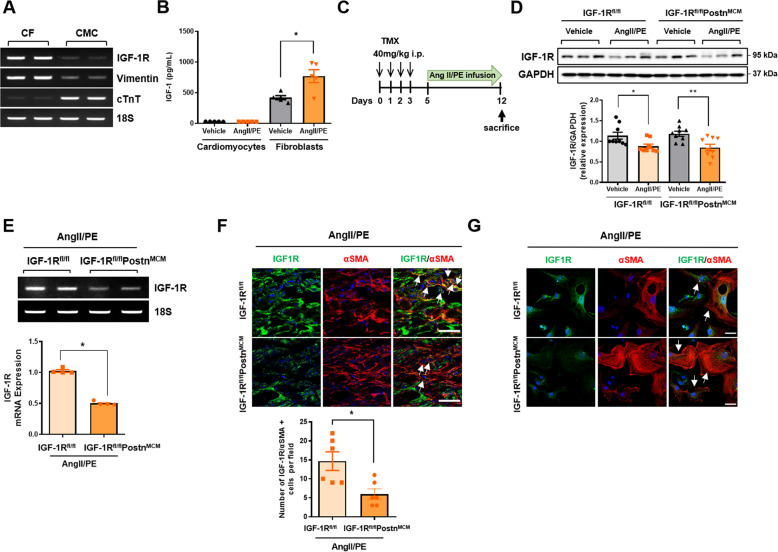
Inducible myofibroblast-specific deletion of IGF-1R in mouse heart. **A** Semiquantitative RT-PCR analysis of *Igf-1r* mRNA expression in C57BL6 mouse cardiac fibroblasts and cardiomyocytes, characterized by vimentin and cardiac troponin T (cTnT), respectively. **B** Cardiomyocytes and fibroblasts were isolated from AngII/PE-infused C57BL6 WT hearts and cultured for 72 h. IGF concentrations in culture media were measured by ELISA. *n* = 5/group. **C** Schematic diagram of the experimental procedure. WT (IGF-1R^fl/fl^) or CFIGF1RKO (IGF-1R^fl/fl^Postn^MCM^) mice were administered tamoxifen intraperitoneally for four consecutive days. From day 5, the vehicle or AngII/PE mixture was infused by an osmotic minipump for 7 days, and the hearts were harvested. **D** Western blot analysis (*upper panel*) and densitometric ratios (*lower panel*) of IGF-1R. Group sizes: WT vehicle (*n* = 9), WT AngII/PE (*n* = 9), CFIGF1RKO vehicle (*n* = 9), CFIGF1RKO AngII/PE (*n* = 9). **E** Expression of *Igf-1r* mRNA in differentiated cardiac fibroblasts from AngII/PE-infused WT or CFIGF1RKO hearts. Representative RT-PCR is shown (*upper panel*). Relative levels of mRNA expression were normalized with respect to the levels of 18S and the mRNA level of WT hearts was arbitrarily set as 1. *n* = 4/group. **F** Double immunostaining of myocardial cryosections of AngII/PE-infused WT and CFIGF1RKO mice with anti-IGF-1R (green) and anti-αSMA (red) antibodies (*upper panel*) and quantification (*lower panel*). Nuclei were stained with DAPI (blue). Merged images are shown (magnification, ×40, scale bar, 50 µm). To quantify IGF-1R/αSMA-positive cells (*arrows*), a number of αSMA-expressing cells were counted based on the cells expressing IGF-1R. Group sizes: WT AngII/PE (*n* = 6), CFIGF1RKO AngII/PE (*n* = 6). **G** Representative immunostaining for IGF-1R (green), αSMA (red) in isolated cardiac myofibroblasts from AngII/PE-infused WT and CFIGF1RKO mice. *Arrows* indicate stains of IGF-1R/αSMA. Nuclei were stained with DAPI (blue). Merged images are shown (magnification, ×40, scale bar, 25 µm). Data are presented as the mean ± SEM. ^*^P < 0.05; ^**^P < 0.01.

Firstly, we examined the levels of IGF-1R in cardiac fibroblasts compared with cardiomyocytes isolated from mouse hearts using Langendorff perfusion. Surprisingly, semi-quantitative RT-PCR analysis revealed higher expression of IGF-1R mRNA in cardiac fibroblasts than in cardiomyocytes (Fig. 1A). To assess the effects of fibrotic agonists on IGF-1/IGF-1R expression in cardiac cells, we infused C57BL6 mice with AngII and phenylephrine (AngII/PE) through osmotic minipumps for 7 days and then isolated and plated cardiomyocytes and cardiac fibroblasts. IGF-1 secretion was significantly increased in fibroblasts isolated from AngII/PE-infused mice compared with those isolated from vehicle-infused mice (Fig. 1B). By contrast, cardiomyocytes isolated from vehicle-infused or AngII/PE-infused mice secreted relatively low concentrations of IGF-1, suggesting that myofibroblast-derived IGF-1 may be involved in agonist-induced cardiac fibrosis.

Next, we sought to characterize the role of IGF-1R in the development of cardiac fibrosis. For this purpose, male CFIGF1RKO mice (8–10 weeks of age) were administered tamoxifen to mark activated fibroblasts followed by a 7-day infusion with fibrotic agonists AngII/PE and then harvested (Fig. 1C). AngII/PE significantly suppressed IGF-1R expression in the heart, suggesting that IGF-1/IGF-1R signaling may be protective against cardiac injury (Fig. 1D). Although IGF-1R expression was decreased in both AngII/PE-infused wild-type (WT) and CFIGF1RKO hearts, a 51% lower *Igf-1r* mRNA expression was noted in activated cardiac fibroblasts and myofibroblasts from AngII/PE-infused CFIGF1RKO mice than in those from AngII/PE-infused WT mice (Fig. 1E). Furthermore, IGF-1R immunoreactivity was significantly decreased in myofibroblasts, as indicated by co-immunofluorescence for αSMA in myocardial cryosections (Fig. 1F) and isolated myofibroblasts (Fig. 1G), confirming the myofibroblast-specific ablation of IGF-1R.

### Genetic ablation of IGF-1R in myofibroblasts accelerates cardiac fibrosis in response to Ang II/PE infusion

**Fig. 2:**
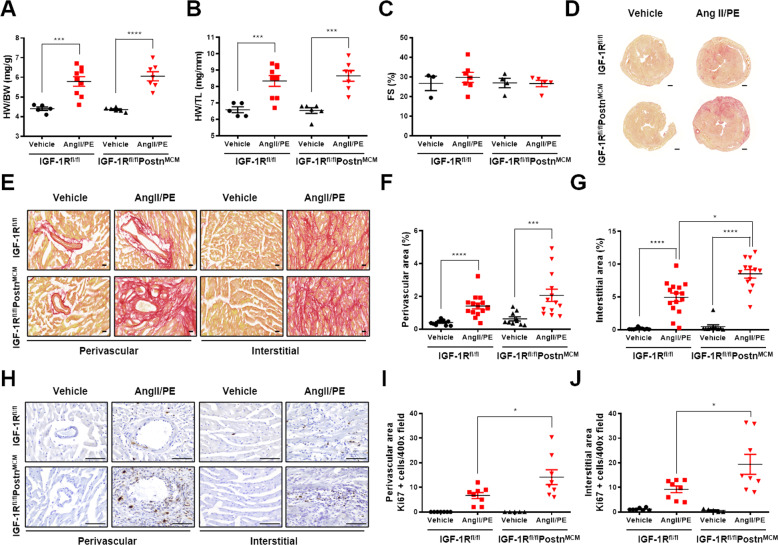
Genetic ablation of IGF-1R in myofibroblast aggravates AngII/PE-induced cardiac fibrosis. **A** Heart weight to body weight (HW/BW) ratio. **B** Heart weight to tibia length (HW/TL) ratio. Group sizes: WT vehicle (*n* = 5), WT AngII/PE (*n* = 9), CFIGF1RKO vehicle (*n* = 6), CFIGF1RKO AngII/PE (*n* = 7). **C** Echocardiographic analysis of fractional shortening (FS). Group sizes: WT vehicle (*n* = 3), WT AngII/PE (*n* = 7), CFIGF1RKO vehicle (*n* = 4), CFIGF1RKO AngII/PE (*n* = 5). (**D**) Representative Sirius red staining of heart cryosections from vehicle or AngII/PE-infused mice (magnification, ×1.7, 500 µm). **E** Representative Sirius red staining of the perivascular and interstitial areas (magnification, ×40, 20 µm). Quantification of perivascular fibrosis (**F**) and interstitial fibrosis (**G**). Group sizes: WT vehicle (*n* = 10), WT AngII/PE (*n* = 15), CFIGF1RKO vehicle (*n* = 10), CFIGF1RKO AngII/PE (*n* = 13). **H** Representative images of Ki67 immunostaining (magnification, ×40, 100 µm). Quantification of Ki67-positive perivascular cells (**I**) and interstitial cells (**J**). Group sizes: WT vehicle (*n* = 7), WT AngII/PE (*n* = 8), CFIGF1RKO vehicle (*n* = 5), CFIGF1RKO AngII/PE (*n* = 8). Data are presented as the mean ± SEM. ^*^P < 0.05; ^***^P < 0.001; ^****^P < 0.0001.

AngII/PE infusion for 7 days did not alter body weight (BW) but significantly increased heart weight, heart weight (HW) to BW ratios, and HW to tibia length (TL) ratios in both genotypes (Fig. 2A, B; Supplementary material online, Fig. S1A and S1B). In addition, both WT and CFIGF1RKO mice showed preserved cardiac contractile function as demonstrated by comparable fractional shortening after AngII/PE infusion (Fig. 2C; Supplementary material online, Table S1). These findings suggest that deletion of IGF-1R in myofibroblasts did not affect the degree of cardiac hypertrophy or contractile function.

**Fig. 3:**
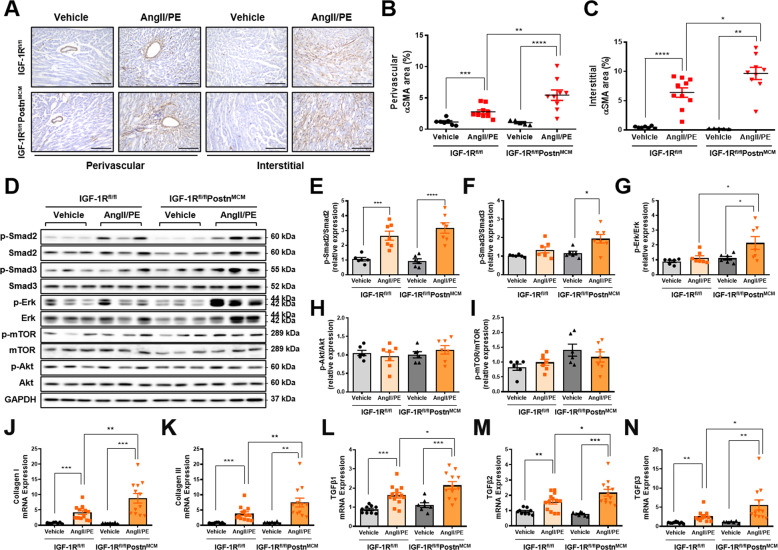
Genetic ablation of IGF-1R in myofibroblast expands αSMA expression and promotes activation of Erk signaling in response to AngII/PE. **A** Representative images of αSMA immunostains (magnification, ×20, 200 µm) at the perivascular and interstitial areas. Quantification of αSMA-positive perivascular (**B**) and interstitial (**C**) areas. Group sizes: WT vehicle (*n* = 7), WT AngII/PE (*n* = 10), CFIGF1RKO vehicle (*n* = 5), CFIGF1RKO AngII/PE (*n* = 9). Western blot analysis (**D**) and densitometric ratios of phosphorylated Smad2 (**E**), phosphorylated Smad3 (**F**), phosphorylated Erk (**G**), phosphorylated Akt (**H**), and phosphorylated mTOR (**I**). Group sizes: WT vehicle (*n* = 6), WT AngII/PE (*n* = 6–7), CFIGF1RKO vehicle (*n* = 6), CFIGF1RKO AngII/PE (*n* = 6–7). mRNA quantification of collagen I (**J**), collagen III (**K**), *Tgfβ1* (**L**), *Tgfβ2* (**M**), and *Tgfβ3* (**N**). Results were normalized to 18 S and mRNA levels in the vehicle were arbitrarily set as 1. Group sizes: WT vehicle (*n* = 9), WT AngII/PE (*n* = 13), CFIGF1RKO vehicle (*n* = 7), CFIGF1RKO AngII/PE (*n* = 12). Data are presented as the mean ± SEM. ^*^P < 0.05; ^**^P < 0.01; ^***^P < 0.001; ^****^P < 0.0001.

Next, the Sirius red staining was performed to detect cardiac fibrosis. Myocardial collagen deposition was higher in AngII/PE infused CFIGF1RKO mice than in AngII/PE-infused WT mice (Fig. 2D). Mice subjected to AngII/PE infusion had higher perivascular and interstitial fibrosis than vehicle-infused mice (Fig. 2E–G). Interstitial fibrosis but not perivascular fibrosis was significantly higher in AngII/PE-infused CFIGF1RKO hearts than in AngII/PE-infused WT hearts. During activation, fibroblasts rapidly proliferate and upregulate cell cycle genes [3]. To detect proliferating cells, the sections were immunostained for Ki67, a marker of the cell cycle. AngII/PE-infused CFIGF1RKO hearts showed a dramatic increase in Ki67-positive cells in both perivascular and interstitial areas relative to AngII/PE-infused WT mice hearts (Fig. 2H–J). Ki67-positive cells were primarily myofibroblasts (Supplementary material online, Fig. S3A–3C). Furthermore, as illustrated in Fig. 3A, B, αSMA, expressions of myofibroblasts differentiation marker, was significantly higher in the perivascular area in AngII/PE-infused CFIGF1RKO mice than in AngII/PE-infused WT mice. In addition, there was a significantly larger interstitial αSMA-positive area in AngII/PE-infused CFIGF1RKO than in AngII/PE-infused WT mice (Fig. 3C). Although Smad2 phosphorylation was upregulated in both genotypes, phospho-Smad3 and phospho-Erk levels were significantly increased in AngII/PE-infused CFIGF1RKO hearts compared with those in AngII/PE-infused WT hearts (Fig. 3D–I). Moreover, collagen I, collagen III, *Tgfβ1, Tgfβ2*, and *Tgfβ3* mRNA expression levels were significantly higher in AngII/PE-infused CFIGF1RKO hearts than in AngII/PE-infused WT hearts (Fig. 3J–N). Thus, we conclude that IGF-1R in myofibroblasts is essential for limiting cardiac fibrosis in agonists-infused mice.

### Low dose IGF-1 attenuates Ang II-induced cardiac fibrosis irrespective of cardiac hypertrophy

**Fig. 4:**
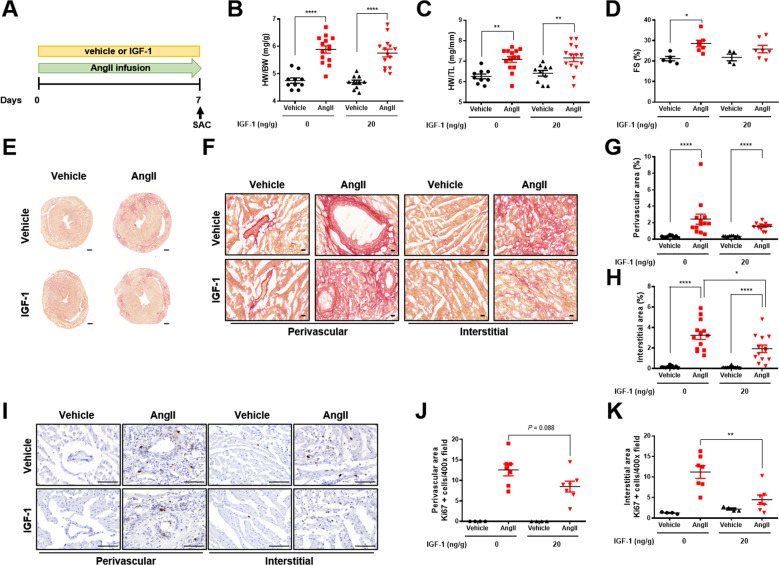
Systemic infusion of low dose IGF-1 attenuates AngII-induced cardiac fibrosis. **A** Schematic diagram of the experimental procedure. IGF-1 (20 ng/g/day) or vehicle was co-infused with AngII (3 µg/g/day) via a subcutaneously implanted osmotic minipump for 7 days in C57BL6 mice. **B** HW/BW ratio. Group sizes: vehicle (*n* = 10), AngII (*n* = 14), vehicle+IGF-1 (*n* = 10), AngII+IGF-1 (*n* = 14). **C** HW/TL ratio. Group sizes: vehicle (*n* = 10), AngII (*n* = 14), vehicle+IGF-1 (*n* = 10), AngII+IGF-1 (*n* = 14). **D** Echocardiographic analysis of fractional shortening (FS). Group sizes: vehicle (*n* = 5), AngII (*n* = 7), vehicle+IGF-1 (*n* = 4), AngII+IGF-1 (*n* = 7). **E** Representative Sirius red staining of hearts from AngII-infused mice treated with or without IGF-1. **F** Representative Sirius red staining of the perivascular and interstitial areas (magnification, x40, 20 µm). Quantification of perivascular fibrosis (**G**) and interstitial fibrosis (**H**). Group sizes: vehicle (*n* = 9), AngII (*n* = 13), vehicle+IGF-1 (*n* = 9), AngII+IGF-1 (*n* = 13). **I** Representative images of Ki67 immunostaining (magnification, ×40, 100 µm). Quantification of Ki67-positive perivascular cells (**J**) and interstitial cells (**K**). Group sizes: vehicle (*n* = 4), AngII (*n* = 7), vehicle+IGF-1 (*n* = 4), AngII+IGF-1 (*n* = 7). Data are presented as the mean ± SEM. ^*^P < 0.05; ^**^P < 0.01^; ****^P < 0.0001.

**Fig. 5:**
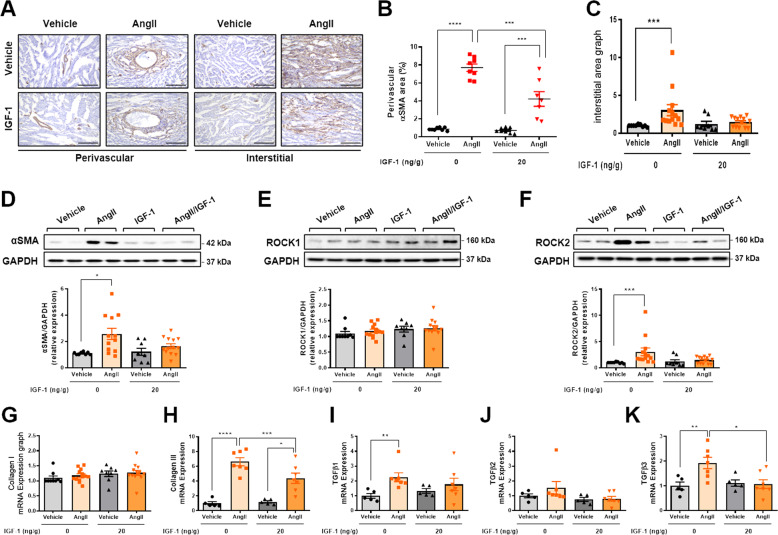
Decreased myofibroblast differentiation and fibrosis-associated genes in IGF-1-treated fibrotic hearts. **A** Representative images of αSMA immunostaining (magnification, ×20, 200 µm) at perivascular and interstitial area. Quantification of αSMA-positive perivascular (**B**) and interstitial (**C**) area. Group sizes: vehicle (*n* = 8), AngII (*n* = 8), vehicle+IGF-1 (*n* = 8), AngII+IGF-1 (*n* = 7). Western blot analysis and the densitometric ratio of αSMA (**D**), ROCK1 (**E**), and ROCK2 (**F**). Group sizes: vehicle (*n* = 8–9), AngII (*n* = 12–13), vehicle+IGF-1 (*n* = 8), AngII+IGF-1 (*n* = 13). mRNA quantification of collagen I (**G**), collagen III (**H**), *Tgfβ1* (**I**), *Tgfβ2* (**J**), and *Tgfβ3* (**K**). Results were normalized to 18 S, and mRNA levels in vehicle were arbitrarily set as 1. Group sizes: vehicle (*n* = 5), AngII (*n* = 7), vehicle+IGF-1 (*n* = 5), AngII+IGF-1 (*n* = 7). Data are presented as the mean ± SEM. ^*^P < 0.05; ^**^P < 0.01; ^***^P < 0.001; ^****^P < 0.0001.

To determine whether IGF-1 had potential therapeutic effects on cardiac fibrosis, continuous low dose IGF-1 (20 ng/g/day) was co-infused in AngII-administrated mice for 7 days (Fig. 4A). Administration of AngII decreased BW to the same extent in both the genotypes (Supplementary material online, Fig. S2A and 2B). In addition, HW/BW and HW/TL were comparable between mice treated with AngII/vehicle and with AngII/IGF-1 (Fig. 4B, C). Although AngII infusion induced a similar degree of cardiac hypertrophy, fractional shortening, which increased in mice treated with AngII/vehicle, was not increased in AngII/IGF-1-infused mice compared with that in AngII/vehicle-infused mice (Fig. 4D; Supplementary material online, Table S2). Moreover, IGF-1 treatment markedly attenuated AngII-induced interstitial fibrosis (Fig. 4E–H). Notably, Ki67-positive cells colocalized to αSMA-positive cells (Supplementary Material Online, Fig. S4A and S4B) suggesting that IGF-1 suppressed cardiac fibroblast proliferation in AngII-administered mice (Fig. 4I–K). Furthermore, IGF-1 treatment suppressed AngII-induced myofibroblast differentiation, as indicated by decreased αSMA expression (Fig. 5A–D). In cardiac fibroblasts, rho-associated coiled-coil containing kinase (ROCK)2 is necessary to cause cardiac hypertrophy and fibrosis [10]. Indeed, expression of ROCK2 but not ROCK1 was increased in AngII-infused hearts and was suppressed by IGF-1, supporting a crucial role of ROCK2 in the antifibrotic effects of IGF-1 (Fig. 5E, F). Moreover, IGF-1 significantly suppressed AngII-induced collagen and *Tgfβ3* mRNA expression (Fig. 5G–K). Infusion of AngII over 7 days increased systolic blood pressure, which was not affected by IGF-1 treatment, indicating that IGF-1 had blood pressure-independent antifibrotic effects (Supplementary material online, Fig. S5). These results show that IGF-1 treatment alleviates the progression of cardiac fibrosis in an established mouse model.

### Regulation of the inhibitory effects of IGF-1 on AngII-induced cardiac fibrosis by Akt-, and ROCK2-dependent signaling pathways

**Fig. 6:**
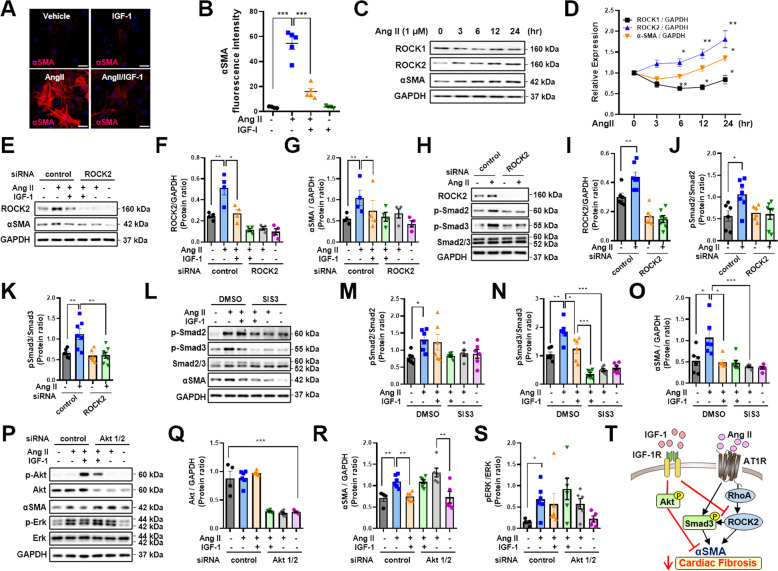
Inhibitory effects of IGF-1 on AngII-induced differentiation of cardiac fibroblasts into myofibroblasts. **A** Adult rat cardiac fibroblasts were treated with AngII (1 µM) or AngII plus IGF-1 (100 ng/mL) for 24 h, and immunocytochemical staining was performed. Representative images of αSMA and DAPI (magnification, ×40, 25 µm) and **B** quantification of fluorescence intensity. *n* = 4–7/group. Cells were treated with AngII for 3–24 h. The lysates were then immunoblotted with the indicated antibodies. Western blot analysis (**C**) and densitometric ratios (**D**) of ROCK1, ROCK2, and αSMA. *n* = 4–6. Cells were transfected with 30 nM negative control or 30 nM ROCK2 siRNA for 24 h and then incubated with vehicle or IGF-1 with AngII for 24 h. Western blot analysis (**E**) and densitometric ratios (**F**, **G**). *n* = 4 each. Knockdown of ROCK2 was performed in cardiac fibroblasts and treated with AngII for 6 h. Western blot analysis (**H**) and densitometric ratios (**I**, **K**). *n* = 6–7/group. Cardiac fibroblasts were treated with vehicle or the Smad3 inhibitor SIS3 (5 μM) for 40 min and then incubated with vehicle or IGF-1 with AngII for 24 h. Western blot analysis (**L**) and densitometric ratios (**M–O**). *n* = 6/group. Cardiac fibroblasts were transfected with 10 nM negative control or 5 nM Akt1 and Akt2 siRNA for 24 h and then incubated with vehicle or IGF-1 with AngII for 24 h. Western blot analysis (**P**) and densitometric ratios (**Q–S**). *n* = 4–6/group. Data are presented as the mean ± SEM. ^*^P < 0.05; ^**^P < 0.01^; ***^P < 0.001. **T** Schematic diagram depicting the roles of IGF-1 signaling in modulating AngII-induced cardiac fibrosis.

IGF-1 inhibited myofibroblast differentiation, as demonstrated by decreased αSMA protein expression in AngII-treated adult rat cardiac fibroblasts (Fig. 6A, B). To explore the role of ROCK2, we stimulated cells with AngII for 0–24 h. Notably, the expression levels of ROCK2 protein but not ROCK1 protein were time-dependently increased, accompanied by the upregulation of αSMA (Fig. 6C, D). To verify whether αSMA is a downstream target of ROCK2, we transfected ROCK2 siRNA into rat cardiac fibroblasts and treated them with AngII up to 24 h. As shown in Fig. 6E–G, IGF-1 significantly suppressed AngII-induced upregulation of ROCK2 and aSMA as well. However, as a consequence of the knockdown of ROCK2, AngII-induced upregulation of αSMA was not observed, confirming that AngII-regulated αSMA expression through ROCK2. These data suggested that ROCK2 was a target of IGF-1 in AngII-induced cardiac fibroblast differentiation. In addition, treatment with ROCK2 siRNA inhibited AngII-induced phosphorylation of Smad2 and Smad3, indicating that ROCK2 was a critical target for regulating the fibrotic gene program (Fig. 6H–K). Although AngII phosphorylated both Smad2 and Smad3, treatment with IGF-1 inhibited phosphorylation of Smad3 but not Smad2. Moreover, the addition of the pharmacologic Smad3 inhibitor SIS3 blunted AngII-induced αSMA, suggesting that IGF-1 could directly or indirectly inhibit the activity of Smad3 which is located between AngII and αSMA (Fig. 6L–O). Finally, to determine whether IGF-1-Akt signaling regulated αSMA expression, we knocked down both Akt1 and Akt2 by transfection with Akt1 and Akt2 siRNA into cardiac fibroblasts. As shown in Fig. 6P–S, Akt1 and Akt2 knockdown represses the inhibitory effect of IGF-1 on AngII-induced αSMA expression. Based on the observation that phospho-Erk was increased in AngII/PE-infused CFIGF1RKO hearts, we also measured Erk phosphorylation. Phospho-Erk was significantly increased after AngII stimulation, whereas the addition of IGF-1 to AngII did not affect Erk phosphorylation, indicating that Erk signaling was not likely involved in mediating the AngII/αSMA pathway. Taken together, these results suggested that IGF-1 inhibited AngII-induced cardiac fibrosis through Akt-dependent and ROCK2- associated pathways (Fig. 6T).

## Discussion

In the current study, we demonstrated a novel function of cardiac fibroblast IGF-1 signaling in the development of cardiac fibrosis. IGF-1 is mainly synthesized and secreted in the liver; however, some reports have shown that IGF-1 is also produced by cardiac fibroblasts upon exposure to pressure overload stimuli [5]. Here, we found that fibrotic agonists stimulated IGF-1 production from differentiated cardiac fibroblasts, which may be a compensatory response against cardiac injury. Moreover, via its cognate receptor IGF-1R in cardiomyocytes, fibroblast-derived IGF-1 may have essential roles in myocardial protection against pressure overload in mice [11].

Most studies on the role of cardiac IGF-1 signaling have focused on cardiomyocytes. Based on our previous reports, deletion of IGF-1R in cardiomyocytes contributes to abnormal cardiac remodeling, such as blunted hypertrophic response to exercise training [6] or delayed aging-associated myocardial pathologies [7]. However, to date, the roles of autocrine/paracrine IGF-1 signaling in cardiac fibroblasts against agonist-induced fibrosis remains elusive. In this study, we observed a higher expression of IGF-1R in cardiac fibroblasts than in cardiomyocytes. Moreover, decreased IGF-1R expression was observed in AngII/PE-infused hearts. This prompted us to investigate the protective role of cardiac fibroblast IGF-1R against injury-induced cardiac fibrosis. We showed that CFIGF1RKO hearts exhibited a more severe degree of fibrosis and myofibroblast differentiation than WT hearts in the AngII/PE infusion model. Thus, in fibroblasts, IGF-1R expression may counteract fibrosis against cardiac injury. Notably, CFIGF1RKO hearts exhibited higher Smad3 and Erk phosphorylation in response to AngII/PE stimuli. However, Smad2 phosphorylation, which is known to be increased by AngII treatment [12], was not significantly affected by IGF-1R deletion in myofibroblasts. Moreover, Akt was not involved in cardiac remodeling in our model. Thus, crosstalk between Smad3 and Erk signaling [13, 14] is likely to be involved in accentuated activation of fibrosis in AngII/PE-treated CFIGF1RKO mice. Taken together, these findings may account for injury-induced cardiac fibrosis that can be controlled by modifying the IGF-1/IGF-1R signaling.

Myocardial interstitial fibrosis is characterized by excessive accumulation of collagen in the ECM and it is a common finding of non-ischemic cardiomyopathy such as hypertensive heart disease [2]. Importantly, the degree of interstitial fibrosis is associated with cardiovascular death in patients with heart failure. A recent study has demonstrated that mitogen-activated protein kinase (MAPK) p38α deletion in myofibroblasts reduces the fibrotic response to ischemia injury [15]. Thus, it is prudent to explore strategies to inhibit fibroblast differentiation for the effective treatment of cardiac fibrosis. In terms of the phenotype conversion in critical evolution of cardiac fibrosis, cardiac fibroblasts proliferate and differentiate into secretory phenotype cells, e.g., myofibroblasts, which can be driven by many stimuli including mechanical force, TGFβ, connective tissue growth factor (CTGF), and renin–angiotensin–aldosterone system (RAAS) activation. AngII is known to induce collagen synthesis through the AT1 receptor (AT1R)/TGFβ/Smads-signaling pathway in cardiac fibroblasts [16]. Clinical studies have shown that both ACEI lisinopril [17] and ARB losartan [18] reduce interstitial fibrosis in patients with hypertensive heart disease, independent of their anti-hypertensive effects. However, these drugs modestly regress cardiac fibrosis. Although clinical trials in patients with heart failure with preserved ejection fraction with pirfenidone, a TGFβ inhibitor approved for idiopathic pulmonary fibrosis, are ongoing [19], it remains to be established whether these drugs confer an additional anti-fibrotic benefit over ACEIs or ARB [20]. Furthermore, AngII stimulates TGFβ production in cardiac fibroblasts and myofibroblasts and is required for AngII-induced cardiac hypertrophy and fibrosis [1, 21]. In addition, AngII can rapidly activate the Smad2/3 pathway independent of TGFβ activation during epithelial–mesenchymal transdifferentiation [22]. In the current study, we observed that IGF-1 treatment suppressed AngII-induced TGFβ3 expression, indicating that IGF-1 plays important role in suppressing AngII-induced TGFβ signaling. Thus, the involvement of the AngII/TGFβ/SMAD2 axis in the differentiation of fibroblasts into myofibroblasts may be much more complex and warrants further investigation.

IGF-1 has potential cardioprotective effects following cardiac injury through both pro-survival and anti-apoptotic mechanisms [23]. For example, low-dose (600 pg) intracoronary IGF-1 administered following reperfusion in a porcine model of myocardial infarction was shown to have acute pro-survival effects on the myocardium [24]. In addition, one-time skeletal muscle injection of adeno-associated virus 5 encoding IGF-1 in coronary occlusion heart failure rat model significantly reduced not only cardiomyocyte apoptosis but myocardial fibrosis [25]. However, the roles of IGF-1 in fibrosis are still controversial. Acromegalic patients exhibit myocardial hypertrophy and interstitial fibrosis depending on the duration of growth hormone and IGF-1 excess [26]. Recent studies have shown that short-term IGF-1 treatment (1 μg/g/day) using an osmotic minipump for 3 days after acute myocardial infarction improves cardiac function by modulating the acute inflammatory phase but does not alleviate fibrosis [27]. We speculate that the beneficial effects of IGF-1 on cardiac fibrosis may vary depending on the treatment duration and dosing. Given that low-dose IGF-1 resulted in promising outcomes in the porcine study, we conducted a preclinical study with a nanomolar concentration of IGF-1. In addition, we provide a rationale to explain why continuous infusion may be an effective mode of drug delivery.

Although several studies examined the link between IGF-1 signaling and organ fibrosis, the precise mechanism is still obscure. IGF-1 induces senescence and limits fibrosis in a p53-dependent manner in hepatic stellate cells [28]. IGF-1 protects against tubular fibrotic changes induced by renal injury via activation of the Erk pathway [29]. One novelty of the current study is the elucidation of the molecular basis for IGF-1 function during AngII stimulation. ROCK1 and ROCK2 are members of the serine/threonine-protein kinase family, which mediates the downstream effects of the small GTP-binding protein RhoA [30]. Excess activity of ROCKs can lead to the development of cardiovascular diseases. Recent studies have shown that the ROCK2 pathway in fibroblasts is critically important for mediating AngII-stimulated cardiac hypertrophy and fibrosis [10]. The nonselective ROCK inhibitor fasudil reduces the development of cardiac fibrosis in AngII-treated mice [31]. In the current study, AngII decreased the expression of ROCK1 in adult rat cardiac fibroblasts. Notably, cardiomyocyte-specific ROCK1 deficiency promotes pressure overload-induced cardiac hypertrophy and fibrosis, whereas cardiomyocyte-specific ROCK2-deficient mice show alleviation of cardiac hypertrophy and fibrosis under exposure to pressure overload, suggesting that inhibition of ROCK1 is not a suitable therapeutic strategy for heart failure [32]. Future studies using myofibroblast-specific ROCK1 KO mice may reveal the opposite roles of ROCK1 and ROCK2 in fibroblast differentiation. We demonstrated crosstalk between IGF-1 and AngII at ROCK2, suggesting that the IGF-1/ROCK2 cascade is responsible for protection from AngII-induced cardiac fibrosis. Previous studies have shown crosstalk between IGF-1R and AT1R via upregulation of their reciprocal receptors in response to IGF‐1 or AngII stimulation in smooth muscle cells [33]. By contrast, our study demonstrated that IGF-1R signaling antagonized AngII signaling in cardiac fibroblasts. Additional studies are warranted to further elucidate the cross-relationships between IGF-1R and AT1R in cardiac fibroblasts.

Canonical TGFβ signaling occurs via phosphorylation of Smad2 and Smad3. Notably, cardiac fibroblast-specific deletion of Smad3 but not Smad2 significantly attenuates the cardiac fibrotic response to pressure overload [34]. Our study is the first to investigate the activation of Smad3 in fibroblasts in the context of AngII stimulation. Consequently, IGF-1 alleviates AngII-mediated cardiac fibrosis via its ability to inhibit Smad3 phosphorylation. Additionally, Akt phosphorylation is required for IGF-1 action to inhibit αSMA expression. Although TGFβ is known to induce a non-canonical response via the PI3K/Akt, RhoA-ROCK, and MAPK cascades during differentiation of cardiac fibroblasts [35], no changes in Akt phosphorylation were found in AngII/PE-infused mouse hearts or AngII-treated cardiac fibroblasts in this study. Further studies are needed to fully elucidate the profibrotic pathways mediated by TGFβ or AngII.

In conclusion, our findings demonstrate that IGF-1 is a critical regulator of cardiac fibrosis and could be a potential therapeutic agent for the management of AngII-associated cardiac fibrosis.

### Limitations of the study

This study had several limitations. First, although we clearly demonstrated that IGF-1 attenuated cardiac fibrosis in an AngII-infusion mouse model, cardiac fibrosis is already present in pathological conditions, such as hypertension or myocardial infarction, when antifibrotic treatment is started in humans. Thus, future studies should address whether IGF-1 treatment significantly reverses established cardiac fibrosis in complementary models. Second, we did not measure blood IGF-1 concentrations or systolic blood pressure in CIGF1RKO mice in the current study.

## Materials and methods

### Mouse models

A tamoxifen-inducible myofibroblast-specific IGF-1R KO (CFIGF1RKO: IGF-1R^fl/fl^Postn^MCM^) mouse model was generated by crossing mice homozygous for floxed IGF-1R alleles in which loxP sites flank exon 3 of the IGF-1R gene [6] with mice containing a tamoxifen-inducible Cre recombinase (MerCreMer) expression cassette within the Postn genetic locus (PostnMCM) [36]. The PostnMCM knock-in mice were obtained from Dr. Jeffery Molkentin (Cincinnati Children’s Hospital Medical Center, USA). All mice were maintained on a C57BL6J background. Tamoxifen was dissolved in corn oil at a final concentration of 4 mg/mL and injected intraperitoneally into mice for 4 consecutive days at a dose of 40 μg/g BW using a 27-gauge needle. We injected the same amount of tamoxifen to wildtype (WT: IGF-1R^fl/fl^) mice to exclude the confounding results from tamoxifen-induced cardiac toxicity [37]. To induce periostin-expressing cardiac myofibroblasts, we used an established AngII/PE-infusion model, as previously described [36]. Periostin is expressed by injury-induced activated cardiac fibroblasts but not expressed in quiescent fibroblasts. Under 1.5% isoflurane anesthesia, osmotic minipumps were implanted subcutaneously, delivering 1.2 μg/g/day AngII (Sigma, St. Louis, MO, USA; cat. no. A9525; 50 mg) and 35 μg/g/day PE HCl (Sigma; cat. no. P6126-5G) for 7 days. Control animals were infused with saline. Mice were randomly assigned to each infusion group. Random assignment was based on a table generated by PASS^TM^ 11 software (NCSS, Kaysville, UT, USA). The randomization code was generated by a statistician who was not otherwise involved in the study. Exclusion criteria of experimental mice were infection after minipump implantation. In the second experiment, male C57BL6 mice (8–10 weeks of age) were randomly assigned to the four osmotic minipump infusion groups: (1) vehicle infusion, (2) AngII infusion, (3) IGF-1 infusion, and (4) AngII plus IGF-1 infusion. The mice were treated for 7 days before harvesting hearts. The animals were fed standard chow and housed in temperature-controlled, pathogen-free facilities with a 12 h light/dark cycle. No blinding was used. In vivo experiments included at least three mice per group. The sample sizes are sufficient to determine whether there is a meaningful difference among experimental groups according to previous studies performed by our laboratory. All animal experiments were conducted following guidelines approved by the Institutional Animal Care and Use Committee of Chung-Ang University; the investigation conforms with the Guide for the Care and Use of Laboratory Animals published by the US National Institutes of Health. The mice were not subjected to any invasive or metabolic studies other than echocardiography.

### Echocardiography

Mice were anesthetized with isoflurane via inhalation (3% for induction and 1.5% for maintenance of anesthesia) using a laboratory animal anesthesia machine (VetEquip, Vivermore, CA, USA). Echocardiography was performed using the Vevo 770 System (VisualSonics Inc., Toronto, Ontario, Canada) with a 30-MHz transducer in the 2-dimensional and M-mode images.

### Blood pressure measurements

Noninvasive systolic blood pressure was measured by tail-cuff methodology using the BP-2000 Blood Pressure Analysis System (Visitech Systems). The occlusion cuff was placed at the base of the tail and the volume–pressure recording sensor cuff was placed adjacent to the occlusion cuff. Mice were trained for 2 days, then consecutive measurements of 15–20 times were recorded. The average of the measured values over 3 days was used in the analysis [38].

### Isolation of cardiomyocytes and fibroblasts from adult mice or rats

Mice or rats were euthanized by inhalation of 3–5% isoflurane. Cardiomyocytes and cardiac fibroblasts were isolated using retrograde Langendorff perfusion, as previously described [39]. Fifteen-gauge (rats) and 20-gauge (mice) needles were inserted into the aorta, and the hearts were immediately perfused with an EDTA buffer for 5 min and perfusion buffer for 5 min. Subsequently, the hearts were perfused with enzyme solution (collagenase type II [Worthington; cat. no. LS004176]; 1.2 mg/mL/protease [Sigma; cat. no. P5147; 0.08 mg/mL]) for 45 min (rats) or 10 min (mice) and then separated. The enzyme solution was neutralized using a 2% fetal bovine serum stop solution, and the cell suspension was filtered through a 100 μm cell strainer. The filtered cell suspension was collected and centrifuged at 300×*g* for 10 min. After centrifugation, the supernatant (noncardiomyocyte fraction) was collected for incubation of fibroblasts, and the cell pellet was resuspended in calcium reintroduction buffer for incubation of cardiomyocytes. Isolated cardiomyocytes were plated in laminin-coated dishes and allowed to attach for 3 h, and the growth medium was then added. For cultures of adult rat cardiac fibroblasts, noncardiomyocyte fractions were added and incubated in a growth medium, with medium changes every 2–3 days in 100 mm dishes to eliminate cellular debris and endothelial cells. Cardiac fibroblasts were then seeded in 35 or 60 mm dishes. For cultures of adult mice cardiac fibroblasts, noncardiomyocyte fractions were seeded in 60 mm dishes, the medium was changed after 1 day, and cells were then cultured for 72 h for collection of culture medium for IGF-1 ELISA analysis and immunocytochemistry. The collected culture medium was centrifuged at 13,000 rpm for 10 min at 4 °C, and supernatants were used for IGF-1 ELISA (cat. no. MG100; R&D Systems, USA). All in vitro experiments were performed at least three times.

### Histological analysis

Following deep anesthesia with 3% isoflurane, the mice were sacrificed by cervical dislocation. The tissue preparation and staining were performed as described previously [40–42]. Cardiomyocytes and cardiac fibroblasts from adult mice or cardiac fibroblasts from adult rats were seeded into culture plates with coverslips. Cells were fixed with 20% MeOH for 15 min at −20 °C. Cells were then permeabilized for 10 min with 0.1% Tween-20 in phosphate-buffered saline (PBS) and blocked with 10% bovine serum albumin (BSA) blocking solution in PBS for 1 h at room temperature. Cells were incubated with primary antibodies (anti-IGF1R [Santa Cruz Biotechnology, Dallas, TX, USA; cat. no. sc-713; dilution, 1:50]; anti-αSMA [Sigma-Aldrich; cat. no. A5288; dilution, 1:100]) overnight at 4 °C. The next day, cells were washed three times for 10 min each and then incubated with fluorescent-labeled secondary antibodies (Alexa Fluor goat anti-rabbit or anti-mouse IgG) for 1 h at room temperature. Cells were washed for 10 min in PBS, and coverslips were mounted with DAPI solution. Images were obtained using a confocal microscope (LSM 700; Carl Zeiss). For histological analysis of mouse heart tissues, heart tissues were fixed in 4% paraformaldehyde, incubated in 15–30% sucrose overnight at 4 °C, and mounted in OCT compound at −20 to −80 °C. Frozen sections (6 μm) were washed in TDW for removal of the OCT compound. Sirius Red staining was performed by incubation in Bouin’s solution for 1 h at 65 °C and 0.1% Sirius Red solution for 1 h at room temperature. For immunohistochemical analysis of Ki67 and αSMA, peroxidase activity was blocked with 3% H_2_O_2_ in MeOH for 20 min at room temperature. Slides were then incubated with blocking solution (5% BSA) for 1 h and treated with primary antibodies (anti-Ki67 [Abcam, Cambridge, UK; cat. no. ab16667; dilution, 1:50]; anti-αSMA [Cell Signaling Technology, Danvers, MA, USA; cat. no. 19245; dilution, 1:50]) for 45 min at 37 °C. In addition, samples were incubated with biotinylated secondary antibodies and avidin-biotin complex reagents (Vector Laboratories; cat. no. PK6100) for 30 min at room temperature. Chromogenic detection was performed using a DAB Substrate Kit. For immunofluorescence analysis of IGF-1R, αSMA, and Ki67, slides were permeabilizated with 0.3% PBS-T for 15 min at room temperature and then incubated with 10% BSA for 1 h and primary antibodies (anti-IGF-1R [Santa Cruz Biotechnology; cat. no. sc-713; dilution, 1:50]; anti-αSMA [Cell Signaling Technology; cat. no. 19245; dilution, 1:200]; anti-Ki67 [eBioscience; cat. no. 14-5698-82; dilution, 1:50]) overnight at 4 °C. Samples were then labeled with Cy3-conjugated anti-αSMA [Sigma-Aldrich; cat. no. C6198; dilution, 1:200] antibody, goat anti-rabbit IgG (H + L)-Alexa Fluor 488, and goat anti-rat IgG (H + L)-Alexa Fluor 568. Slides were washed for 15 min in PBS, and coverslips were mounted with DAPI solution. Images were obtained using a confocal microscope (LSM 700; Carl Zeiss). The number of positively stained cells was calculated in each of six mice per group using Image J software.

### Western blot analysis

Heart tissues or cells from mice were lysed with lysis buffer (20 mM Tris–HCl, pH 7.4; 1% Triton X-100; 1 mM EDTA; 30 mM HEPES; 50 mM Na_4_P_2_O_7_; 100 mM NaF) containing 1× Protease Inhibitor Cocktail (Roche Molecular Biochemicals, Indianapolis, IN, USA) and phosphatase inhibitors. Lysates were incubated on ice for 15 min and centrifuged at 13,000 rpm at 4 °C for 10 min. The supernatants were used for western blot analysis. Protein quantification was performed using BCA assays, and proteins were separated by sodium dodecyl sulfate-polyacrylamide gel electrophoresis on 8–10% gels. Proteins were transferred to polyvinylidene difluoride membranes and blocked. Primary antibodies were diluted in blocking solution (5% BSA in TBST) according to the manufacturer’s protocol (anti-IGF-1R [Santa Cruz Biotechnology; cat. no. sc-713; dilution, 1:500]; phospho-SMAD3 [Abcam; cat. no. ab52903; dilution, 1:1500]; anti-SMAD3 [Sigma-Aldrich; cat. no. ZRB1005; dilution, 1:1000 for mouse samples]; phospho-SMAD2 [Cell Signaling Technology; cat. no. 3108; dilution, 1:1000]; anti-SMAD2/3 [Cell Signaling Technology; cat. no. 8685; dilution, 1:1000]; anti-phospho-ERK [Cell Signaling Technology; cat. no. 9101; dilution, 1:2000]; anti-ERK [Cell Signaling Technology; cat. no. 9102; dilution, 1:2000]; anti-phospho-mammalian target of rapamycin [mTOR; Cell Signaling Technology; cat. no. 2974; dilution, 1:1000]; anti-mTOR [Cell Signaling Technology; cat. no. 2972; dilution, 1:1000]; anti-phospho-AKT [Cell Signaling Technology; cat. no. 4060; dilution, 1:1000]; anti-AKT [Cell Signaling Technology; cat. no. 9272; dilution, 1:1000]; anti-αSMA [Cell Signaling Technology; cat. no. 19245; dilution, 1:2000]; anti-ROCK1 [Cell Signaling Technology; cat. no. 4035; dilution, 1:1000]; anti-ROCK2 [Cell Signaling Technology; cat. no. 9029; dilution, 1:1000]; and anti-glyceraldehyde 3-phosphate dehydrogenase [Cell Signaling Technology; cat. no. 2118; dilution, 1:2000]). Membranes were then incubated with secondary antibodies in 5% skim milk for 1 h at room temperature, and protein bands were visualized using EzWestLumi ECL solution. Densitometric quantification was performed using Bio-Rad Image Lab software (Bio-Rad Laboratories, Hercules, CA, USA).

### RNA isolation and semi-quantitative or quantitative RT-PCR analysis

Total RNA was obtained from the heart tissues or cells using the RiboEx Total RNA Solution. First-strand cDNA was synthesized from 1 μg total RNA with random primers using a 20 μL reverse transcription system (Thermo Scientific; cat. no. K1622). The cDNA was then used for semiquantitative or quantitative RT-PCR. PCR was performed using EmeraldAmp PCR Master Mix (TaKaRa, Shiga, Japan) for semiquantitative RT-PCR and SsoFast EvaGreen Supermix (Bio-Rad LAboratories) for quantitative PCR. Semi-quantitative RT-PCR condition was as follows: 94 °C for 5 min followed by 27 cycles at 94 °C for 30 s, 60 °C for 30 s, 72 °C for 45 s, and a final extension at 72 °C for 7 min. The quantitative RT-PCR condition was as follows: 95 °C for 2 min followed by 35 cycles at 95 °C for 10 s, 56 °C for 10 s, 72 °C for 15 s, and then melting curves were analyzed. The 18S rRNA was used as a normalization gene. Primers are listed in Table S3. To assess the specificity of the amplified PCR products, a post-amplification melting curve analysis was performed, and relative quantification was calculated using the comparative cycle threshold method.

### Statistical analysis

Data are presented as the mean ± SEM. For intergroup comparisons, the distribution of the data was firstly evaluated for normality using the Shapiro–Wilk test, Shapiro–Wilk test after log-transformation, and q–q plot. The normally distributed data were compared using a *t*-test or two-way ANOVA. Non-normally distributed data were analyzed using either a Mann–Whitney *U*-test or Mann–Whitney *U*-test with adjusting the α-level by Bonferroni inequality. For serial data, within-group difference was analyzed with the linear mixed-effects model (LMEM). Levene’s test for homogeneity of variances was performed. *P* < 0.05 was considered statistically significant. All analyses were conducted using the Statistical Package for the Social Sciences software suite (version 23; IBM Corp., Armonk, NY, USA).

## Supplementary information

Ock_Supplementary
