# Supplementary material for: IGF-1 protects against angiotensin II-induced cardiac fibrosis by targeting αSMA
Source: Cell Death Dis. 2021 Jul 9;12(7):688. doi: 10.1038/s41419-021-03965-5 (PMC8270920; doi:10.1038/s41419-021-03965-5)
Supplement: Supplementary file 1 — Ock_Supplementary [file 41419_2021_3965_MOESM1_ESM.docx]

**Supplementary Data**

**Article Title: IGF-1 protects against angiotensin II-induced cardiac fibrosis by targeting αSMA**

**Authors: Sangmi Ock^1^, Woojin Ham^1^, Chae Won Kang^1^, Hyun Kang^2^, Wang Soo Lee^3^*, Jaetaek Kim^1^***

**^1^Division of Endocrinology and Metabolism, Department of Internal Medicine, College of Medicine, Chung-Ang University, Seoul, Korea**

**^2^ Department of Anesthesiology, College of Medicine, Chung-Ang University, Seoul, Korea**

**^3^ Division of Cardiology, Department of Internal Medicine, College of Medicine, Chung-Ang University, Seoul, Korea**

**Contents:**

**Supplementary Figure Legends**

**Supplementary Figure S1.**

**Supplementary Figure S2.**

**Supplementary Figure S3.**

**Supplementary Figure S4.**

**Supplementary Figure S5.**

**Supplementary Table S1**

**Supplementary Table S2**

**Supplementary Table S3**

**Supplementary Figure Legends**

**Figure S1.** Body weight (**A**) and heart weight (**B**) from vehicle or AngII/PE-infused mice. Group sizes : WT vehicle (n=5), WT AngII/PE (n=9), CFIGF1RKO vehicle (n=6), CFIGF1RKO AngII/PE (n=7). Data are presented as the mean ± SEM.

**Figure S2.** Body weight difference before (**A**) and after (**B**) infusion from AngII infused mice treated with or without IGF-1. Group sizes : vehicle (n=10), AngII (n=14), vehicle+IGF-1 (n=10), AngII/IGF-1 (n=14). Data are presented as the mean ± SEM.

**Figure S3. (A)** Representative images of αSMA/Ki67-positive myofibroblasts (magnification, x20, scale bar, 100µm). Quantification of αSMA and Ki67-positive perivascular (**B**) and interstitial (**C**) areas. To quantify αSMA/Ki67-positive cells (*arrows*), the number of αSMA expressing cells was counted based on the merged cells with Ki67 and DAPI. Group sizes: WT AngII/PE (n=6), CFIGF1RKO AngII/PE (n=6). Data are presented as the mean ± SEM. ^*^*P* < 0.05

**Figure S4. (A)** Representative images of αSMA/Ki67-positive myofibroblasts (magnification, x20, scale bar, 100µm). Quantification of αSMA/Ki67-positive perivascular (**B**) and interstitial (**C**) areas. To quantify αSMA/Ki67-positive cells (*arrows*), the number of αSMA expressing cells was counted based on the merged cells with Ki67 and DAPI. Group sizes: AngII (n=6), AngII+IGF-1 (n=6). Data are presented as the mean ± SEM. ^*^*P* < 0.05

**Figure S5.** Tail cuff systolic blood pressure from AngII-infused mice treated with or without IGF-1. Group sizes: vehicle (n=6), AngII (n=5), vehicle+IGF-1 (n=6), AngII+IGF-1 (n=6). Data are presented as the mean ± SEM. **P* < 0.05; ***P* < 0.01.

**Supplementary Figure S1.**


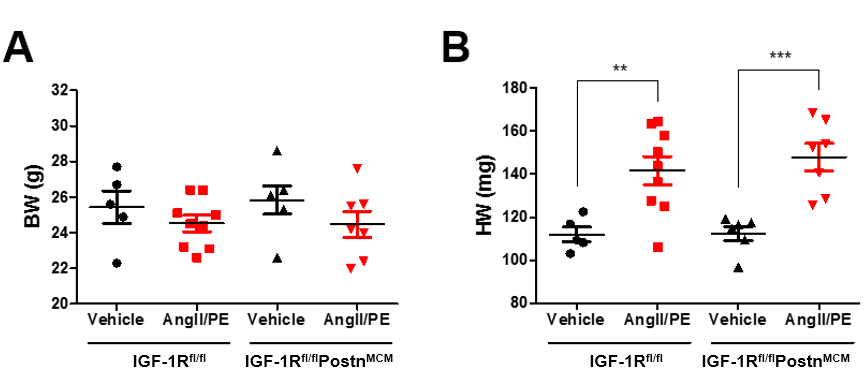


**Supplementary Figure S2.**

**A B**

**Supplementary Figure S3.**


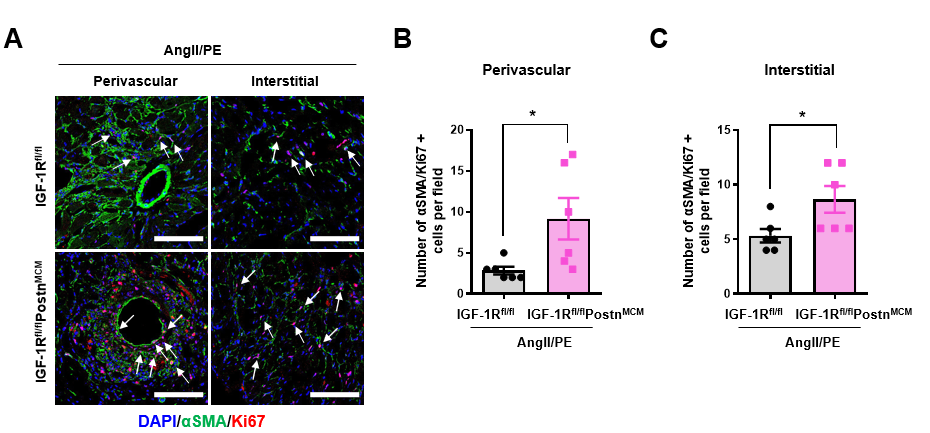


**Supplementary Figure S4.**


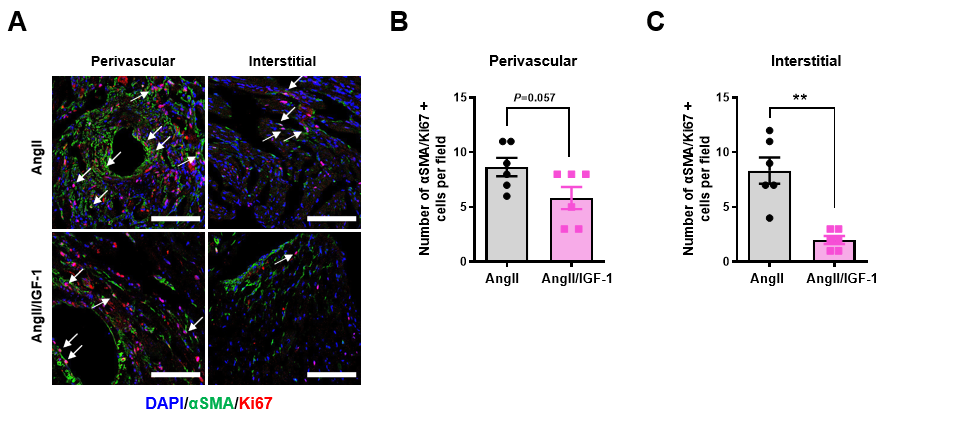


**Supplementary Figure S5.**


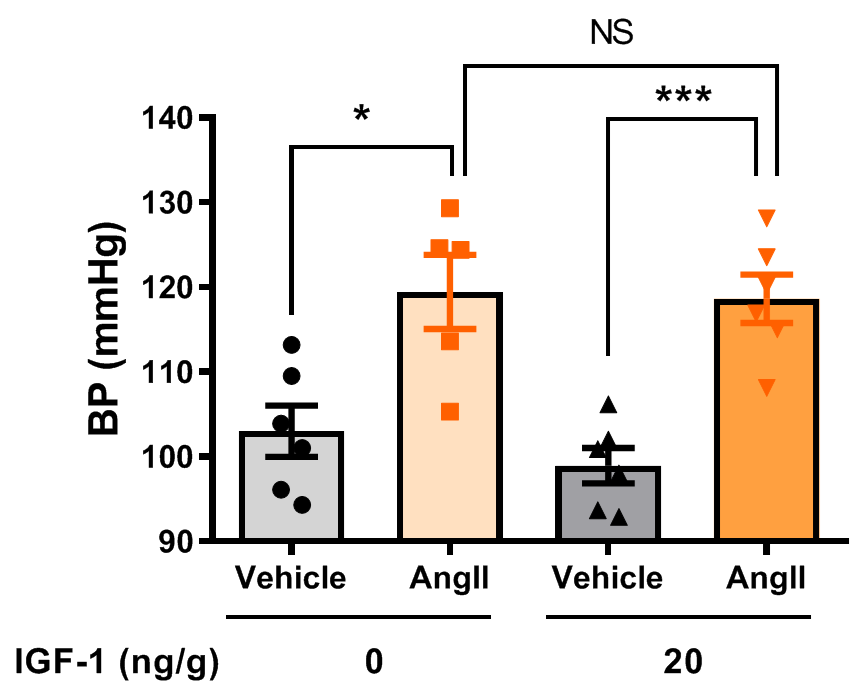


**Supplementary Table S1**


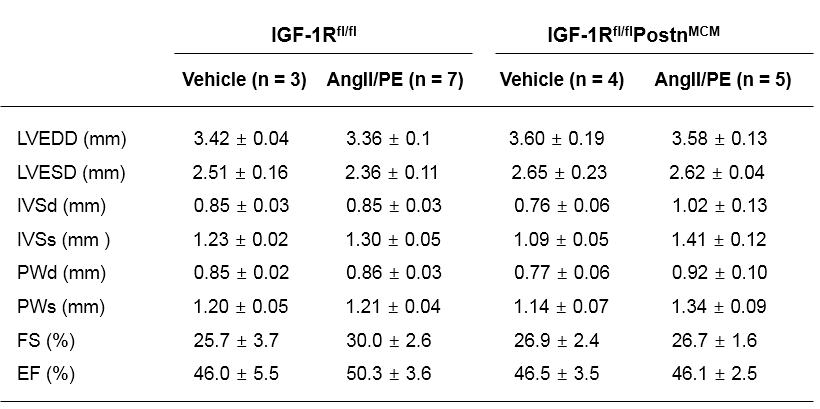


**Table S1.** Echocardiographic analysis 7 days after vehicle or AngII/PE infusion. Values are mean ± SEM. ^**^*P* < 0.01 versus young WT. LVEDD indicates left ventricular end diastolic diameter; LVESD, left ventricular end systolic diameter; IVSd, interventricular septum thickness measured in diastole; IVSs, interventricular septum thickness measured in systole; PWd, left ventricular posterior wall thickness measured in diastole; PWs, left ventricular posterior wall thickness measured in systole; FS, fractional shortening; EF, ejection fraction.

**Supplementary Table S2**


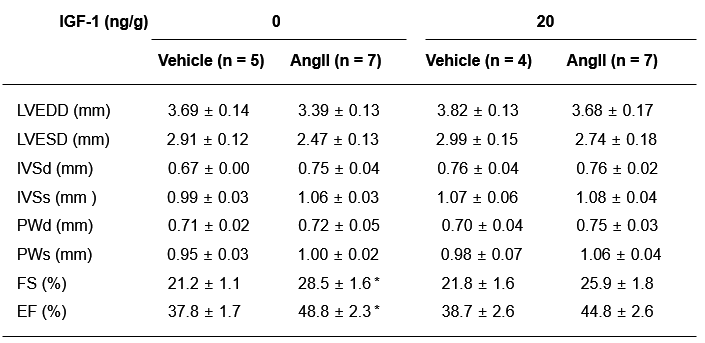


**Table S2.** Echocardiographic analysis 7 days after vehicle or AngII with or without IGF-1 infusion. Values are mean ± SEM. ^**^*P* < 0.05 versus young WT. LVEDD indicates left ventricular end diastolic diameter; LVESD, left ventricular end systolic diameter; IVSd, interventricular septum thickness measured in diastole; IVSs, interventricular septum thickness measured in systole; PWd, left ventricular posterior wall thickness measured in diastole; PWs, left ventricular posterior wall thickness measured in systole; FS, fractional shortening; EF, ejection fraction.

**Supplementary Table S3. Primers for semi-quantitative or quantitative RT-PCR of mouse genes.**

**(1) Semi-quantitative RT-PCR**

| ***18S*** | forward | 5′- AAG CAT TTG CCA AGA ATG TTT T -3′ |
| --- | --- | --- |
|  | reverse | 5′- ATG CCA GAG TCT CGT TCG TTA T -3′ |
| ***Ctnt*** | forward | 5′- ACC AAC TGA GAG AGA AGG CC -3′ |
|  | reverse | 5′- GCA CAG CTT TGA CGA GAA CA -3′ |
| ***Vimentin*** | forward | 5′- CGC TTT GCC AAC TAC ATC GA -3′ |
|  | reverse | 5′- CCT CCT GCA ATT TCT CTC GC -3′ |
| ***Igf-1r*** | forward | 5′- CCC AGA GCA TGT ACT GTA TCC C -3′ |
|  | reverse | 5′- CAC AGC TTG GGA TTG AAA GCA A -3′ |

**(2) Quantitative RT-PCR**

| ***18S*** | forward | 5′- CGA AAG CAT TTG CCA AGA AT -3′ |
| --- | --- | --- |
|  | reverse | 5′- AGT CGG CAT CGT TTA TGG TC -3′ |
| ***Collagen I*** | forward | 5′- ACT GGT ACA TCA GCC CGA AC -3′ |
|  | reverse | 5′- TAC TCG AAC GGG AAT CCA TC -3′ |
| ***Collagen III*** | forward | 5′- ACC AAA AGG TGA TGC TGG AC -3′ |
|  | reverse | 5′- GAC CTC GTG CTC CAG TTA GC -3′ |
| ***Tgfβ1*** | forward | 5′- GAC TCT CCA CCT GCA AGA CC -3′ |
|  | reverse | 5′- GAC TGG CGA GCC TTA GTT TG -3′ |
| ***Tgfβ2*** | forward | 5′- AGC TAC CTG GGT CCA TTC CT -3′ |
|  | reverse | 5′- GAC GCA GAA AAG GCT GAA AC -3′ |
| ***Tgfβ3*** | forward | 5′- GAT GAG CAC ATA GCC AAG CA -3′ |
|  | reverse | 5′- TTT CCA GAC CCA AGT TGG AC -3′ |
